# End-of-Life Care Received by Physicians Compared With Nonphysicians

**DOI:** 10.1001/jamanetworkopen.2019.7650

**Published:** 2019-07-24

**Authors:** Hannah Wunsch, Damon Scales, Hayley B. Gershengorn, May Hua, Andrea D. Hill, Longdi Fu, Therese A. Stukel, Gordon Rubenfeld, Robert A. Fowler

**Affiliations:** 1 Department of Anesthesia, University of Toronto, Toronto, Ontario, Canada; 2 Interdepartmental Division of Critical Care Medicine, University of Toronto, Toronto, Ontario, Canada; 3 Department of Critical Care Medicine, Sunnybrook Health Sciences Centre, Toronto, Ontario, Canada; 4 Department of Anesthesiology, Columbia University, New York, New York; 5 ICES, Toronto, Ontario, Canada; 6 Institute of Health Policy, Management and Evaluation, University of Toronto, Toronto, Ontario, Canada; 7 Department of Medicine, University of Toronto, Toronto, Ontario, Canada; 8 Division of Pulmonary, Critical Care, and Sleep Medicine, University of Miami Miller School of Medicine, Miami, Florida; 9 Division of Critical Care Medicine, Albert Einstein College of Medicine, Bronx, New York; 10 Department of Epidemiology, Mailman School of Public Health, Columbia University, New York, New York; 11 The Dartmouth Institute for Health Policy and Clinical Practice, Geisel School of Medicine at Dartmouth, Hanover, New Hampshire

## Abstract

**Importance:**

The idea that physicians as patients choose less-aggressive care at the end of life for themselves is an often-cited rationale to advocate for less technology-laden end-of-life care.

**Objective:**

To assess end-of-life care received by physicians compared with nonphysicians in a system with universal health care.

**Design, Setting, and Participants:**

In this population-level decedent cohort study of data from April 1, 2004, through March 31, 2015 (fiscal years 2004-2014), in Ontario, Canada, 2507 physicians were matched approximately 1:3 to 7513 nonphysicians (ie, individuals who never were registered as a physician with the College of Physicians and Surgeons of Ontario) according to age, sex, income quintile, and location of residence.

**Main Outcomes and Measures:**

The primary outcome was location of death. Other outcomes included measures of health care use in the last 6 months of life. Differences were assessed using Poisson regression with robust error variances, adjusting for the Charlson Comorbidity Index.

**Results:**

In total, 2516 physicians and 954 836 nonphysicians died between April 1, 2004, and March 31, 2015, in Ontario; 2247 physicians (89.3%) and 474 182 nonphysicians (49.7%) were men. The median (interquartile range) age at death was 82 (74-87) years for the physicians and 80 (68-87) years for the nonphysicians. After matching, data for 2507 physicians and 7513 nonphysicians were analyzed. For physicians, the risk of death at home was no different from that for nonphysicians (42.8% vs 39.0%; adjusted relative risk [aRR], 1.04; 95% CI, 0.99-1.09), but the risk of death in an intensive care unit was increased (11.9% vs 10.0%; aRR, 1.22; 95% CI, 1.08-1.39). In the prior 6 months, physicians had a decreased risk of an emergency department visit (73.0% vs 78.4%; aRR, 0.96; 95% CI, 0.94-0.98) but increased risks of an intensive care unit admission (20.8% vs 19.1%; aRR, 1.14; 95% CI, 1.05-1.24) and of receipt of palliative care services (52.9% vs 47.4%; aRR, 1.18; 95% CI, 1.13-1.23). Among a subgroup of 457 physicians and 1347 nonphysicians with cancer, the risk of death at home or intensive care unit was increased (37.6% vs 28.6%; aRR, 1.30; 95% CI, 1.13-1.50), as was the risk of receiving chemotherapy in the last 6 months of life.

**Conclusions and Relevance:**

There was no difference overall for physicians compared with nonphysicians in terms of the likelihood of dying at home; physicians were more likely to die in an intensive care unit and to receive chemotherapy, but also to receive palliative care services. These findings suggest that physicians do not consistently opt for less-aggressive care but instead receive end-of-life care that includes both intensive and palliative care. These findings inform a more nuanced perspective of what physicians may perceive to be optimal care at the end of life.

## Key Points

**Question:**

What are the patterns of care at the end of life for physicians compared with nonphysicians?

**Findings:**

In this cohort study of 2507 physicians and 7513 nonphysicians who died in Ontario, Canada, physicians were no more likely to die at home than nonphysicians. Overall, they did not consistently opt for less-aggressive care but instead used both intensive and palliative care more than nonphysicians.

**Meaning:**

These findings highlight a more nuanced perspective of what physicians may perceive to be optimal care at the end of life.

## Teaser

This cohort study compares the location of death and measures of health care use in the last 6 months of life for physicians vs nonphysicians in Canada.

## Introduction

Matching end-of-life care to patients’ stated preferences by reducing technology-laden, hospital-based treatments is a common focus of quality improvement in many developed countries.^1,2,3,4,5^ Studies^6,7,8^ have, therefore, focused on identifying factors that are associated with more-intensive treatment before death, such as age, socioeconomic status, race, geoethnic origin, and religion.

High treatment intensity at the end of life is well documented in North America,^3,9^ even among patients who may have previously stated preferences for less-aggressive care. When physicians become patients and approach the end of life themselves, there is a common but unproven perception within the health care community that doctors opt for less-aggressive end-of-life care on the basis of their own experience and knowledge of the potential downsides of technology-laden institution-focused care. Much of the focus of end-of-life care has been on location of death, because it has been observed that many individuals in developed countries die in acute care hospitals and nursing homes, despite expressed preferences for dying at home.^3,4,5^ Moreover, death at home may reflect access to substantial support, such as home palliative care and hospice services.^10,11^ Two studies from the United States,^12,13^ focused primarily on location of death, found very slight decreases in the likelihood of a hospital-based death for physicians. However, addressing this question in a system with universal health coverage theoretically minimizes confounding due to individual financial concerns and variability in access to acute care, allowing for better isolation of the impact of personal choices made by physicians at the end of life. We therefore sought to assess the intensity of treatment received by physicians in comparison with similar nonphysicians (ie, individuals who never were registered as a physician with the College of Physicians and Surgeons of Ontario) who died in Ontario, Canada. The primary outcome was location of death, with the hypothesis that physicians are more likely to receive less-intensive end-of-life care.

## Methods

### Study Setting, Design, and Data Sources

We undertook an observational study of all Ontarians dying between April 1, 2004, and March 31, 2015 (fiscal years 2004-2014), and compared physician with nonphysician decedents aged 24 to 105 years. We identified physicians using the College of Physicians and Surgeons of Ontario database of all physicians registered to practice in Ontario, enabling identification and linkage to the Ontario registered persons database and other commonly linked population health administrative data sets on the basis of full name, sex, and date of birth (and occasionally date of death).^14^ These databases, which are used extensively for research purposes,^14,15^ include the discharge abstract database containing all inpatient health records; the national ambulatory care reporting system containing ambulatory health records; the Office of the Registrar General for Deaths, which includes information on cause of death; and the Ontario drug benefits database, with information on all prescriptions for patients older than 65 years. These data sets were linked using unique encoded identifiers and were analyzed at ICES, an independent, nonprofit research institute funded by an annual grant from the Ontario Ministry of Health and Long-Term Care. For a full list of databases, see eTable 1 in the Supplement. This study was approved by the Research Ethics Board at Sunnybrook Health Sciences Centre, Toronto, Canada. Given the databases used, the need for individual consent was waived. The reporting of this study is compliant with the Strengthening the Reporting of Observational Studies in Epidemiology (STROBE) reporting guideline.^16^

### Data Linkage

Data linkage between the College of Physicians and Surgeons of Ontario database and the health administrative databases was performed in 2 steps. First was exact linkage (surname, first given name, date of birth, and sex). Second was a probabilistic approach using 7 options. To ensure accuracy using this second strategy, we performed a manual duplicate independent review of all additional names identified using the probabilistic matching approach (see eTable 2 in the Supplement for details of matching). We then assessed patient characteristics (age and sex) of physicians in the College of Physicians and Surgeons of Ontario database who were matched or unmatched (eTable 3 in the Supplement). After linkage to identify physicians, we included all individuals who died in the cohort between April 1, 2004, and March 31, 2015 (fiscal years 2004-2014).

### Measures

As a common measure of preferred, less-aggressive care, the primary outcome was location of death (home, other care facility, intensive care unit [ICU], hospital [non-ICU], or other), identified from ambulatory and inpatient health records and the registered persons database.^4,5,10^ For the last 6 months of life, we also assessed the number of emergency department visits, number of acute care hospitalizations, number of days in acute care hospitals, number of admissions to an ICU (all levels), number of days in an ICU, episodes of mechanical ventilation, number of physicians seen, and procedures performed, including placement of a surgical feeding tube, placement of a tracheostomy, dialysis, chemotherapy and radiotherapy (only for patients with cancer), receipt of in-hospital cardiopulmonary resuscitation, and total health system costs of care.^17^ Receipt of these procedures was identified through linkages across the health administrative data sets according to the diagnostic and intervention codes. We assessed receipt of palliative care services across health sectors according to physician billing codes, diagnostic codes, or patient service codes, as described elsewhere.^18^ We also determined the frequency of 1 or more opioid prescriptions (as total morphine milligram equivalents) filled as outpatients in the last 6 months of life for individuals older than 65 years at the time of death, because the Ontario drug benefits database includes comprehensive outpatient medication data only on people older than 65 years. To estimate individual-level health services costs, we used a costing algorithm available at ICES for use with health administrative data sets, as described elsewhere.^19^ All costs were standardized to 2014 Canadian dollars.

### Statistical Analysis

We first described characteristics of physician and nonphysician decedents, including sex and age at time of death. Next, we performed a 1:3 greedy match between physicians and nonphysicians using the variables age at the time of death, sex, quintile of neighborhood income, rural vs urban residence, and provincial health region (local health integration network). This was a post hoc decision because the unmatched cohort demonstrated large imbalances for some demographic characteristics, particularly income, sex, and rural residence at time of death, between physicians and nonphysician decedents; we thought that a stronger approach to ensure comparison with similar individuals who were not physicians would be matching to balance these key covariates. Age was summarized as mean with standard deviation and median with interquartile range, and categorical data were summarized as frequency and percentages. We compared the characteristics between physicians and nonphysicians before and after matching using standardized differences, with a standardized difference less than 0.1 indicating a negligible difference.

Using the matched cohort, we first assessed unadjusted data, comparing outcomes between groups as absolute differences with 95% confidence intervals. We then used Poisson regression with robust error variance, accounting for matching,^20^ and multiple linear regression models to assess the relationship between the exposure (physician vs nonphysician) and binary and continuous outcomes, respectively, adjusting for the Charlson Comorbidity Index (categorized as 0, 1 to 2, 3 to 4, and 5 or greater). For the primary outcome, we assessed each location of death as a separate variable, compared with all other locations. As a sensitivity analysis, we then analyzed the entire (unmatched) cohort, using Poisson regression models, controlling for age, sex, quintile of income, rural residence, geographic region of residence, and Charlson Comorbidity Index (categorized). Overall, 11% of individuals (10% of physicians and 11% of nonphysicians) had no Charlson score and were assigned a value of 0. For the unmatched cohort, the percentage of other missing data was 0.1% for rural residence and 0.6% for income. Complete case analysis (listwise deletion) was used to address missing values.

Overall costs and categories of costs (continuing care sectors, acute care sectors, and outpatient care sectors) were summarized as means and standard deviations and were compared as absolute differences with 95% confidence intervals. We then fitted models using multiple linear regression on the log-transformed costs to provide adjusted regression and expressed these as percentage change in the geometric mean for each cost category. This was calculated as (exp^β^ – 1) × 100, where β is the regression coefficient.

First, we stratified physicians and nonphysicians by age and determined whether there was an association with age on the likelihood of dying at home using the Cochrane-Armitage test for linear trend. Second, to decrease the likelihood of sudden death with no prior morbidity, which would be “unplanned,” we examined a subgroup of patients who were identified as having any 1 of 9 known chronic diseases,^21^ matching physicians 1:3 with nonphysicians who had any 1 (or more) of these diagnoses. Second, we created a matched cohort (1:3) of physicians and nonphysicians with a diagnosis of cancer in the last 6 months of life. For the cancer cohort, we assessed receipt of any chemotherapy or radiotherapy in the last 6 months of life, as well as all other outcomes. Race/ethnicity was not included in any model because accurate information on race/ethnicity was not available.

All analyses were performed using SAS Enterprise Guide statistical software version 7.12 (SAS Institute) and Excel spreadsheet software version 2016 (Microsoft Corp). Two-sided *P* values are reported for all regression analyses and the Cochrane-Armitage test, with *P* < .05 considered statistically significant.

## Results

There were 2516 physicians and 954 836 nonphysicians identified in Ontario health records who died between April 1, 2004, and March 31, 2015. Physician decedents were predominantly male (2274 [89.3%] vs 474 182 [49.7%] among nonphysicians). Age at time of death was slightly older among physicians (median [interquartile range] age, 82 [74-87] years vs 80 [68-87] years for nonphysicians). The most common cause of death in both groups was ischemic heart disease (13.3% vs 13.7%). Quintile of neighborhood income was skewed among physicians, with 46.5% in the highest quintile (vs 17.6% among nonphysicians), and fewer physicians resided in rural locations (8.5% vs 14.8%). After matching, the cohorts of 2507 physicians and 7513 nonphysicians were balanced with regard to age, sex, quintile of neighborhood income, rural residence, and common causes of death, but physicians had fewer comorbidities (Table 1).

**Table 1.** Patient Characteristics of Physician and Nonphysician Decedents 2004 to 2014, for Entire Population (Unmatched) and Then Matched 1:3 (Physicians: Nonphysicians)

Variable	Unmatched			Matched (1:3)		
	Physicians	Nonphysicians	Standardized Mean Difference	Physicians	Nonphysicians	Standardized Mean Difference
Total decedents, No.	2516	954 836		2507	7513	
Age at death, y						
Mean (SD)	79.2 (12.0)	76.5 (14.3)	0.2	79.2 (12.0)	79.2 (12.0)	0
Median (interquartile range)	82 (74-87)	80 (68-87)	0.2	82 (74-87)	82 (74-87)	0
Male, No. (%)	2247 (89.3)	474 182 (49.7)	1.0	2238 (89.3)	6710 (89.3)	0
Quintile of neighborhood income, No. (%)^a^						
1 (lowest)	237 (9.5)	221 735 (23.4)	0.4	237 (9.5)	711 (9.5)	0
2	259 (10.3)	199 117 (21.0)	0.3	259 (10.3)	776 (10.3)	0
3	352 (14.0)	184 244 (19.4)	0.1	352 (14.0)	1056 (14.1)	0
4	494 (19.7)	177 182 (18.7)	0	494 (19.7)	1482 (19.7)	0
5 (highest)	1165 (46.5)	166 782 (17.6)	0.7	1165 (46.5)	3488 (46.4)	0
Rural residence at time of death, No. (%)^a^	214 (8.5)	141 205 (14.8)	0.2	211 (8.4)	627 (8.3)	0
Common causes of death, No. (%)^b^						
Ischemic heart disease	334 (13.3)	130 434 (13.7)	0	334 (13.3)	1114 (14.8)	0
Cancer of lung and bronchus	95 (3.8)	59 905 (6.3)	0.1	95 (3.8)	415 (5.5)	0.1
Dementia and Alzheimer disease	143 (5.7)	56 770 (5.9)	0	143 (5.7)	420 (5.6)	0
Cerebrovascular diseases	142 (5.6)	48 701 (5.1)	0	140 (5.6)	374 (5.0)	0
Chronic lower respiratory tract diseases	40 (1.6)	33 992 (3.6)	0.1	40 (1.6)	285 (3.8)	0.1
Charlson Comorbidity Index, No. (%)						
0	643 (25.6)	230 530 (24.1)	0	639 (25.5)	1576 (21.0)	0.1
1-2	775 (30.8)	260 680 (27.3)	0.1	774 (30.9)	2119 (28.2)	0.1
3-4	453 (18.0)	177 864 (18.6)	0	451 (18.0)	1517 (20.2)	0.1
≥5	645 (25.6)	285 762 (29.9)	0.1	643 (25.6)	2301 (30.6)	0.1

^a^ Missing data for unmatched cohort for income (9 physicians [0.4%] and 5776 nonphysicians [0.6%]) and location of residence (1 physician [0.04%] and 772 nonphysicians [0.1%]).

^b^ From the Ontario Registrar General for Deaths database.

### Location of Death

In the matched cohort, rates of death at home were similar (42.8% for physicians vs 39.0% for nonphysicians; adjusted relative risk [aRR], 1.04; 95% CI, 0.99-1.09). More physicians than nonphysicians died in an ICU (11.9% vs 10.0%; aRR, 1.22; 95% CI, 1.08-1.39) (Table 2). Among physicians, deaths in a hospital (not an ICU) ranged from 12.5% in those younger than 45 years to 37.5% in those aged 85 years and older, whereas rates of death in an ICU were highest in those aged 45 to 64 years (15.5%) and lowest in those aged 85 years and older (7.5%). Similar trends in the location of death across age groups were observed among nonphysicians (eFigure in the Supplement).

**Table 2.** Intensity of End-of-Life Care Measures for Physicians Compared With Nonphysicians (Matched 1:3)

Variable	Physicians (n = 2507)	Nonphysicians (n = 7513)	Absolute Difference (95% CI)	Relative Risk or Adjusted Regression Coefficient (95% CI)^a^	*P* Value
Location of death, No. (%)					
Home	1073 (42.8)	2927 (39.0)	3.8 (1.6 to 6.1)	1.04 (0.99 to 1.09)^b^	.15
Long-term care	213 (8.5)	680 (9.1)	0.6 (0.0 to 1.8)	0.99 (0.85 to 1.14)^b^	.86
Acute care hospital (non-ICU)	791 (31.6)	2696 (35.9)	4.3 (2.2 to 6.5)	0.93 (0.88 to 0.99)^b^	.03
Acute care hospital (ICU)	298 (11.9)	750 (10.0)	1.9 (0.5 to 3.3)	1.22 (1.08 to 1.39)^b^	.002
Other	132 (5.3)	460 (6.1)	0.9 (0.0 to 1.9)	0.81 (0.67 to 0.97)^b^	.03
Last 6 mo of life, No. (%)					
Any emergency department visits	1830 (73.0)	5888 (78.4)	5.4 (3.5 to 7.3)	0.96 (0.94 to 0.98)^b^	.001
Any hospitalization	1679 (67.0)	5303 (70.6)	3.6 (1.5 to 5.7)	1.00 (0.97 to 1.03)^b^	.96
Any ICU admission	521 (20.8)	1434 (19.1)	1.7 (0.0 to 3.5)	1.14 (1.05 to 1.24)^b^	.003
Any mechanical ventilation	345 (13.8)	1011 (13.5)	0.3 (0.0 to 1.9)	1.06 (0.94 to 1.18)^b^	.34
Any dialysis	101 (4.0)	288 (3.8)	0.2 (0.0 to 1.1)	1.16 (0.93 to 1.44)^b^	.18
Surgical feeding tube	106 (4.2)	246 (3.3)	1.0 (0.1 to 1.8)	1.36 (1.09 to 1.70)^b^	.007
Tracheostomy	30 (1.2)	74 (1.0)	0.2 (0.0 to 0.7)	1.29 (0.85 to 1.96)^b^	.23
Cardiopulmonary resuscitation	49 (2.9)	176 (3.3)	0.4 (0.0 to 1.4)	0.84 (0.62 to 1.14)^b^	.27
Any home care visits	1362 (54.3)	3763 (50.1)	4.2 (2.0 to 6.5)	1.13 (1.08 to 1.18)^b^	<.001
Palliative care received	1326 (52.9)	3560 (47.4)	5.5 (3.3 to 7.8)	1.18 (1.13 to 1.23)^b^	<.001
Emergency department visits, mean (SD), No.	1.5 (1.6)	1.8 (1.8)	0.3 (0.2 to 0.3)	−0.17 (−0.24 to −0.10)^c^	<.001
Hospitalizations, mean (SD), No.	1.1 (1.2)	1.2 (1.2)	0.1 (0.0 to 0.1)	0.02 (−0.02 to 0.07)^c^	.36
Total days in the hospital, mean (SD), No.	13.5 (20.7)	14.2 (20.5)	0.7 (0.0 to 1.7)	0.32 (−0.55 to 1.19)^c^	.47
ICU admissions, mean (SD), No.	0.3 (0.7)	0.3 (0.6)	0.0 (0.0 to 0.1)	0.04 (0.01 to 0.07)^c^	.008
Total days in an ICU, mean (SD), No.	1.9 (7.5)	1.7 (6.8)	0.2 (0.0 to 0.6)	0.33 (−0.00 to 0.66)^c^	.05
Episodes of mechanical ventilation, mean (SD), No.	1.3 (7.4)	1.2 (7.1)	0.1 (0.0 to 0.5)	0.19 (−0.14 to 0.52)^c^	.27
Physicians seen, mean (SD), No.	12.9 (9.4)	12.6 (8.9)	0.3 (0.0 to 0.7)	0.90 (0.54 to 1.26)^c^	<.001
Home care visits, mean (SD), No.	38.4 (81.6)	29.9 (68.7)	8.5 (5.3 to 11.8)	9.70 (6.23 to 13.16)^c^	<.001

Abbreviation: ICU, intensive care unit.

^a^ Both adjusted for Charlson Comorbidity Index (categorized as 0, 1 to 2, 3 to 4, and greater than or equal to 5). Each location of death was assessed as a separate model (vs all other locations).

^b^ Relative risk.

^c^ Adjusted regression coefficient.

### Intensity of Treatment in the Last 6 Months of Life

In the 6 months before death, after adjustment for demographic characteristics, the risk of an emergency department visit was decreased for physicians compared with nonphysicians (73.0% vs 78.4%; aRR, 0.96; 95% CI, 0.94-0.98) but the risk of a hospital admission was not (67.0% vs 70.6%; aRR, 1.00; 95% CI, 0.97-1.03). The risk of an ICU admission was increased (20.8% vs 19.1%; aRR, 1.14; 95% CI, 1.05-1.24), as were the odds of receipt of a surgical feeding tube (4.2% vs 3.3%; aRR, 1.36; 95% CI, 1.09-1.70). There was no significant difference in the odds of receiving mechanical ventilation, dialysis, or cardiopulmonary resuscitation (Table 2).

Receipt of palliative care was increased for physicians compared with nonphysicians (52.9% vs 47.4%; aRR, 1.18; 95% CI, 1.13-1.23), as was the percentage of individuals who received 1 or more home care visits (54.3% vs 50.1%; aRR, 1.13; 95% CI, 1.08-1.18). Results were similar when assessing all physician and nonphysician decedents (unmatched), adjusting for differences in individual characteristics (eTable 4 in the Supplement).

### Subgroup Analyses

Among 1375 physicians with an identified chronic illness matched with 4117 nonphysicians, the patterns of care observed were similar to the matched analysis (eTable 5 in the Supplement), although physicians were more likely to die at home (35.2% vs 30.7%; aRR, 1.12; 95% CI, 1.04-1.22). For the 457 physicians identified as having cancer matched with 1347 nonphysicians with cancer, the likelihood of dying at home was also increased (37.6% vs 28.6%; aRR, 1.30; 95% CI, 1.13-1.50) (eTable 6 in the Supplement). Among this cancer cohort, in the last 6 months of life, the risk of receiving chemotherapy was also increased (37.9% vs 29.8%; aRR, 1.28; 95% CI. 1.13-1.46).

### Opioid Use in the Last 6 Months of Life

Among physicians and nonphysicians older than 65 years for whom full prescription medication data were available, the risk of receipt of at least 1 opioid prescription in the last 6 months of life was increased for physicians (48.4% vs 46.6%; aRR, 1.06; 95% CI, 1.01-1.11). The only other aspect of opioid prescribing that showed a difference was the duration of each opioid prescription filled, which was longer for physicians compared with nonphysicians (mean [SD], 13.8 [11.8] days vs 13.1 [11.6] days; regression coefficient, 0.82; 95% CI, 0.01-1.63) (Table 3).

**Table 3.** Opioid Prescriptions in the Last 6 Months of Life for Physicians Compared With Nonphysicians Aged 65 Years and Older (Matched 1:3)

Variable	Physicians (n = 2202)	Nonphysicians (n = 6600)	Absolute Difference (95% CI)	Relative Risk or Adjusted Regression Coefficient (95% CI)^a^	*P* Value
Any opioid prescription filled, No. (%)	1066 (48.4)	3075 (46.6)	1.8 (0.0 to 4.2)	1.06 (1.01 to 1.11)^b^	.02
Opioid prescriptions filled, mean (SD), No.	2.6 (5.2)	2.6 (6.0)	0.1 (0.0 to 0.4)	0.15 (−0.11 to 0.41)^c^	.25
Morphine milligram equivalents of all prescriptions filled, mean (SD), No.	2482.6 (9942.0)	2394.3 (10 395.7)	88.2 (0.0 to 584.3)	210.67 (−270.66 to 692.00)^c^	.39
Among those who filled any prescription					
Opioid prescriptions filled, mean (SD), No.	5.4 (6.4)	5.5 (7.8)	0.1 (0.0 to 0.6)	−0.01 (−0.47 to 0.46)^c^	.98
Morphine milligram equivalents of all prescriptions filled, mean (SD), No.	5128.1 (13 809.3)	5139.0 (14 761.0)	10.9 (0.0 to 1022.6)	118.79 (−862.04 to 1099.61)^c^	.81
Duration of each opioid prescription filled, mean (SD), d	13.8 (11.8)	13.1 (11.6)	0.7 (0.0 to 1.5)	0.82 (0.01 to 1.63)^c^	.05
Daily morphine milligram equivalents of each opioid prescription filled, mean (SD), No.	41.3 (61.7)	39.6 (63.3)	1.7 (0.0 to 6.1)	2.59 (−1.70 to 6.88)^c^	.24

^a^ Both adjusted for Charlson comorbidity index score (categorized as 0, 1 to 2, 3 to 4, and greater than or equal to 5).

^b^ Relative risk.

^c^ Adjusted regression coefficient.

### Costs of Care in the Last 6 Months of Life

Adjusted to 2014 costs, total costs of care were the same for physicians vs nonphysicians (mean [SD] CAN$38 540 [$43 641] vs $37 451 [$42 670]; adjusted percentage change, 6%; 95% CI, −1% to 13%) (Table 4). In the adjusted analyses, long-term care costs were 59% (95% CI, 52%-65%) lower for physicians than for nonphysicians (mean [SD], $2557 [$6650] vs $3913 [$7810]). However, physicians had higher mean (SD) home care costs ($3093 [$6390] vs $2291 [$4816]; adjusted percentage change, 90%; 95% CI, 60%-125%).

**Table 4.** Total Costs of Care (Canadian Dollars) in the Last 6 Months of Life for Physicians Compared With Nonphysicians (Matched 1:3)

Variable	Physicians (n = 2507)	Nonphysicians (n = 7513)	Absolute Difference (95% CI)	Adjusted Regression Coefficient (95% CI)^a^	Difference, % (95% CI)^b^^,^^c^	*P* Value
Total costs, 6 mo, mean (SD), CAN$	38 540 (43 641)	37 451 (42 670)	1089 (0.0 to 3029)	0.06 (−0.01 to 0.12)	6 (−1 to 13)	.09
Select continuing care sectors, mean (SD), CAN$						
Long-term care	2557 (6650)	3913 (7810)	1357 (1016 to 1697)	−0.90 (−1.06 to −0.74)	−59 (−65 to −52)	<.001
Home care	3093 (6390)	2291 (4816)	802 (565 to 1040)	0.64 (0.47 to 0.81)	90 (60 to 125)	<.001
Complex continuing care	3000 (14 000)	2775 (12 630)	224 (0 to 811)	−0.02 (−0.16 to 0.11)	−2 (−15 to 12)	.72
Acute care sectors, mean (SD), CAN$						
Inpatient care	18 050 (33 511)	17 903 (34 270)	146 (0 to 1687)	0.06 (−0.11 to 0.23)	6 (−10 to 26)	.48
Emergency department	827 (831)	945 (882)	118 (79 to 157)	−0.22 (−0.34 to −0.10)	−20 (−29 to −10)	<.001
Select outpatient care sectors, mean (SD), CAN$						
Physician billings	4224 (5187)	3861 (7448)	363 (49 to 678)	0.16 (0.11 to 0.22)	17 (12 to 25)	<.001
Outpatient clinics	1674 (3237)	1412 (2782)	262 (131 to 393)	0.35 (0.19 to 0.50)	42 (21 to 65)	<.001
Drugs or devices	1771 (3179)	1690 (3073)	81 (0 to 221)	−0.03 (−0.13 to 0.06)	−3 (−12 to 6)	.50

^a^ Multiple linear regression using the log transformation of costs. A constant of $1 was added to all costs before log transformation to allow for inclusion of individuals with 0 costs. Models adjusted for Charlson Comorbidity Index (categorized as 0, 1 to 2, 3 to 4, and greater than or equal to 5).

^b^ Exponentiation of the estimate, subtracting 1 and multiplying this result by 100 to provide a percentage change.

^c^ Positive values denote greater physician costs.

## Discussion

In this comparison of end-of-life care received by physicians compared with nonphysicians in Ontario, differences in patterns of care were modest; we found that physicians were no more likely to die at home but had greater use of palliative care services, consistent with the perception that some physicians opt for less-aggressive care. However, physicians were also more likely to die in an ICU and received more intensive care services overall, suggesting that some physicians opt for aggressive care. On most other measures of care, including mechanical ventilation, use of dialysis, and likelihood of being hospitalized, physicians and nonphysicians were similar. In a system with universal health coverage, informed health care decision makers such as physicians do not consistently opt for less-aggressive care across the board, but instead vary in their choices regarding end-of-life care, with increased use of both intensive and palliative care. These data suggest greater complexity in end-of-life decision-making than a dichotomous model of less vs more.

Many of these modest differences were magnified when we assessed individuals with cancer in the last 6 months of life. Both deaths at home and in the ICU were substantially increased for physicians in this group, and the receipt of chemotherapy in the last 6 months of life was markedly higher. Given that this group is most likely to have time to discuss and plan care choices, it may best reflect care that is primarily driven by the patient (ie, physician as patient) rather than surrogate decision makers.

The comparison between physician and similar nonphysician patients is of particular interest because it addresses whether the problem of information asymmetry—when one party has greater knowledge on a subject—leads to different choices for health care at the end of life.^22,23^ Physicians are familiar with prognostication and options for care, have high health literacy, and, in survey studies, state preferences for less-aggressive care than is typically delivered at the end of life.^24,25^ This perception of a knowledge gap of optimal end-of-life care often informs efforts to improve the quality of end-of-life care, with the assumption that improving information asymmetry may shift patient decision-making toward less-aggressive care choices. Yet, care patterns for physicians were not uniformly less aggressive than those of nonphysicians. Furthermore, the observed higher use of palliative and home care by physicians, as well as fewer emergency department visits, may partly be explained by increased access to these services facilitated by their broader network of health care professionals, including colleagues and friends, and arguably better communication between physicians and their care team. These findings suggest that a more nuanced approach that considers patient preferences, available supports, diagnosis, specific medical problems faced, and stage of terminal illness may be more informative to efforts to improve end-of-life care.

Older studies^26^ of physician preferences have focused on asking physicians to predict what they would prefer at the end of life or in different scenarios. In a study surveying physicians and their patients in 1997, physicians were found to express significant differences in preferences for 6 specific treatments if they were terminally ill, suggesting that “physicians as a group differ substantially in their preferences for end-of-life care.”^27^ Similarly, a comparison of physician choice when weighing clinical scenarios changed when the choice was for themselves vs recommendations for a patient.^28^ In a more recent study^12^ from the United States comparing actual physician care at the end of life with nonphysicians, physicians were less likely to receive care in a hospital or in an ICU compared with the general population and also compared specifically with lawyers, a group with similar educational and socioeconomic status. In another US study^13^ focused on place of death, the findings were similar, with physicians slightly less likely to die in a hospital or in any care facility.

Our study assesses actual care received by physicians rather than hypothetical situations and is unique for providing an assessment across a broader range of measures associated with end-of-life care choices, such as receipt of palliative care services, prescriptions for opioids, and comprehensive costs of care, as well as location of death. Moreover, because Ontario provides universal health care and also covers all prescriptions for individuals older than 65 years, differences are less likely to be attributable to access to care or choices influenced by deductibles, co-payments, or variability in insurance plan coverage. However, it is important to note that some aspects of care, such as home health assistance, may still accrue significant out-of-pocket expenses in the Canadian system.

### Limitations

There are a number of limitations with this study. First, we were not able to link a minority of physicians identified in the College of Physicians and Surgeons of Ontario registry. The greatest reason for nonlinkage was a lack of information regarding sex of physicians, which limited the linkage options. Another likely reason is the migration of some physicians out of Ontario before they required health care. Second, we were unable to identify other professionals in the data who may share a strong health literacy with physicians (eg, nurses) in the likelihood of receipt of certain types of care.^12^ We did adjust for sociodemographic characteristics on the basis of quintile of income for neighborhood, which has been shown to be associated with end-of-life choices,^29,30,31^ in addition to rural or urban residence.^32^ However, physicians and nonphysicians may differ in terms of characteristics that predispose to certain types of decisions. For example, physicians may, on average, select toward action and treatment and be unwilling to tolerate uncertainty.^33,34^ Also, aside from receipt of cardiopulmonary resuscitation, we did not have information on specific goals-of-care discussions or limitations on treatment that may have been put in place.^35^ Finally, because more women than men are now in medical school, the physician workforce has begun to change, and the sex of physicians may affect choices^36,37^; the inclusion of a disproportionate number of men may limit the future generalizability of these findings.

## Conclusions

Provision of aggressive treatment at the end of life, particularly death in an acute care hospital, is currently viewed as a negative indicator of the quality of care.^2^ Physicians were not more likely to die at home compared with similar nonphysicians, but had greater use of both intensive care and palliative care services in the 6 months before death. These findings inform a more nuanced perspective of what physicians may perceive to be optimal care at the end of life and suggests that models of care that encompass both options for intensive and palliative approaches at the end of life merit evaluation.

## Notes

Supplement. **eTable 1.** Databases Used to Record Health Care Use and Costs at the End of Life **eTable 2. **Details of Linkage Using a First-pass Deterministic Approach (1), Followed by Probabilistic Approaches (2-8) **eTable 3.** Characteristics of Matched Versus Unmatched Physicians **eTable 4.** Intensity of Care Measures for Physicians Compared With Nonphysicians (Unmatched) **eTable 5.** Intensity of Care Measures for Physicians Compared With Nonphysicians at the End of Life With a Diagnosis of Specific Chronic Comorbidities (Matched 1:3) **eTable 6.** Intensity of Care Measures for Physicians Compared With Nonphysicians at the End of Life With Cancer (Matched 1:3) **eFigure.** Location of Death for Physicians and Nonphysicians, by Age at Death **eReference**
