# Supplementary material for: End-of-Life Care Received by Physicians Compared With Nonphysicians
Source: JAMA Netw Open. 2019 Jul 24;2(7):e197650. doi: 10.1001/jamanetworkopen.2019.7650 (PMC6659139; doi:10.1001/jamanetworkopen.2019.7650)
Supplement: Supplement. — eTable 1. Databases Used to Record Health Care Use and Costs at the End of Life eTable 2. Details of Linkage Using a First-pass Deterministic Approach (1), Followed by Probabilistic Approaches (2-8) eTable 3. Characteristics of Matched Versus Unmatched Physicians eTable 4. Intensity of Care Measures for Physicians Compared With Nonphysicians (Unmatched) eTable 5. Intensity of Care Measures for Physicians Compared With Nonphysicians at the End of Life With a Diagnosis of Specific Chronic Comorbidities (Matched 1:3) eTable 6. Intensity of Care Measures for Physicians Compared With Nonphysicians at the End of Life With Cancer (Matched 1:3) eFigure. Location of Death for Physicians and Nonphysicians, by Age at Death eReference [file jamanetwopen-2-e197650-s001.pdf]

Supplementary Online Content

Wunsch H, Scales D, Gershengorn HB, et al. End-of-life care received by physicians compared with nonphysicians. *JAMA Netw Open*. 2019;2(7):e1967650. doi:10.1001/jamanetworkopen.2019.7650

eTable 1. Databases Used to Record Health Care Use and Costs at the End of Life
eTable 2. Details of Linkage Using a First-Pass Deterministic Approach (1), Followed by Probabilistic Approaches (2-8)
eTable 3. Characteristics of Matched Versus Unmatched Physicians
eTable 4. Intensity of Care Measures for Physicians Compared With Nonphysicians (Unmatched)
eTable 5. Intensity of Care Measures for Physicians Compared With Nonphysicians at the End of Life With a Diagnosis of Specific Chronic Comorbidities (Matched 1:3)
eTable 6. Intensity of Care Measures for Physicians Compared With Nonphysicians at the End of Life With Cancer (Matched 1:3)
eFigure. Location of Death for Physicians and Nonphysicians, by Age at Death
eReference

This supplementary material has been provided by the authors to give readers additional information about their work.

© 2019 Wunsch H et al. *JAMA Network Open.*

eTable 1. Databases Used to Record Health Care Use and Costs at the End of Life

Health care Sector	Database	Description
**Continuing Care**		
Long-term Care	Continuing Care Reporting System (CCRS)	Population-based resident information for over 600 publicly funded residential care homes with 24-hour nursing care
Complex Continuing Care	CCRS	Population-based information for all patients staying in a designated complex continuing care bed. These individuals are typically deemed to be in a nonacute state, but still in need for treatment (e.g., rehabilitation) in an institution
Home Care	Home Care Database (HCD) Resident Assessment Instrument-Home Care (RAI-HC)	Data from the Ontario Association of Community Care Access Centers, responsible for providing all publicly funded home care
Rehabilitation	National Rehabilitation Reporting System (NRS)	Data from participating adult inpatient rehabilitation facilities and programs across Ontario
**Acute Care**		
Inpatient without ICU	CIHI-DAD[a]	Administrative, clinical, and demographic data on all hospital discharges in Ontario
Inpatient with ICU	CIHI-DAD	Individuals with at least one Intensive Care Unit (ICU) visit in their last year of life
Same Day Surgery (SDS)	CIHI-DAD-SDS	Administrative, clinical and demographic data on same day surgeries performed in hospitals in Ontario
Emergency Department	National Ambulatory Care Reporting System (NACRS)	Captures all emergency department visits in Ontario
**Outpatient Care**		
Outpatient clinics	NACRS	Select outpatient visits held in hospitals, including dialysis clinics and cancer care clinics
Physician Billings	Ontario Health Insurance Plan (OHIP) Claims Database	Claims data for physicians in Ontario—includes claims in both inpatient and outpatient settings.
Nonphysician Billings	OHIP	Health professionals for provincially insured services, such as select midwives, oral surgeons, chiropractors, optometrists, and physiotherapists. Some care may occur for inpatients
Laboratory	OHIP	Outpatient laboratory services. Does not include laboratory services for inpatients
Drugs/Devices	Ontario Drug Benefit (ODB), Assistive Devices Program (ADP)	Drugs for those over 65 years, on social assistance, residents of LTC, home care recipients, Trillium drug program and special drugs program recipients for those qualifying for assistance. Select medically-necessary devices including home oxygen and respiratory devices.

[a] CIHI-DAD: Canadian Institute for Health Information-Discharge Abstract Database: http://www.ncbi.nlm.nih.gov/pmc/articles/PMC4374686/pdf/pone.0121759.pdf

© 2019 Wunsch H et al. *JAMA Network Open.*

eTable 2. Details of Linkage Using a First-Pass Deterministic Approach (1), Followed by Probabilistic Approaches (2-8)

**Linkage Pass Number**	**Description of Linkage Variables**	**Matches, N (%)**
Deterministic		
1.	Surname + Given Name 1 + Date of Birth + Sex	52,872 (86.7)
Probabilistic[a]		
2.	Surname Initial + Given Name 1 Initials (1st-3 Chars) + DOB	4,928 (8.1)
3.	DOB + Sex	2,421 (4.0)
4.	Surname Initial + Given Name 1 Initials (1st-3 Chars) + Birth Year + Sex	306 (0.5)
5.	Given Name 1 Initials (1st-3 Chars) + Birth Month + Birth Day + Sex	305 (0.5)
6.	NYSIIS (the phonetic code for surname) + Birth Year + Sex	28 (0.1)
7.	NYSIIS + Birth Year	114 (0.2)
8.	Death Date	43 (0.1)
**Total No. of Matches**		**61,017**
**No. of CPSO Records**		**89,362**
**Match Rate**		**68.3%**

DOB = date of birth; NYSIIS = New York State Identification and Intelligence System; CPSO = College of Physicians and Surgeons of Ontario

[a] included manual review of each probable linkage

© 2019 Wunsch H et al. *JAMA Network Open.*

eTable 3. Characteristics of Matched Versus Unmatched Physicians

	Matched Physicians (n = 61,017)	Unmatched Physicians (n = 28,345)
**Age, mean (SD)**	70.1 (13.7)	67.3 (13.3)
**Sex**		
**Male**	38,422 (63.0)	5,844 (20.6)
**Female**	20,476 (33.6)	3,364(11.9)
**Unknown**	2,119 (3.5)	19,137 (67.5)

© 2019 Wunsch H et al. *JAMA Network Open.*

eTable 4. Intensity of Care Measures for Physicians Compared With Nonphysicians (Unmatched)

	Physicians (n = 2,516)	Nonphysicians, (n = 954,836)	Absolute Difference (95% CI)	Adjusted Relative Risk[a] (95% CI)	P Value
**Location of death, n (%)**					
**Home**	1,079 (42.9)	385,270 (40.3)	2.5 (0.6, 4.5)	1.04 (1.00, 1.09)	0.05
**Long-term care facility**	213 (8.5)	77,015 (8.1)	0.4 (0.0, 1.5)	1.07 (0.94, 1.22)	0.29
**Acute care hospital (non-ICU)**	792 (31.5)	333,248 (34.9)	3.4 (1.6, 5.3)	0.93 (0.88, 0.98)	0.0099
**Acute care hospital (ICU)**	299 (11.9)	97,528 (10.2)	1.7 (0.5, 2.9)	1.21 (1.09, 1.35)	0.0003
**Other**	133 (5.3)	61,775 (6.5)	1.2 (0.2, 2.2)	0.78 (0.66, 0.92)	0.003
**Last 6 months of life**					
**Any ED visits, n (%)**	1,837 (73.0)	738,941 (77.4)	4.4 (2.7, 6.0)	0.96 (0.94, 0.98)	<.0001
**Any hospitalization, n (%)**	1,682 (66.9)	655,276 (68.6)	1.8 (0.0, 3.6)	1.00 (0.98, 1.02)	0.88
**Any ICU admission, n (%)**	523 (20.8)	185,195 (19.4)	1.4 (0.0, 2.9)	1.12 (1.04, 1.20)	0.003
**Any mechanical ventilation, n (%)**	345 (13.7)	132,674 (13.9)	0.2 (0.0, 1.5)	1.04 (0.95, 1.15)	0.40
**Any dialysis, n (%)**	101 (4.0)	33,843 (3.5)	0.5 (0.0, 1.2)	1.24 (1.02, 1.49)	0.03
**Surgical feeding tube, n (%)**	106 (4.2)	29,493 (3.1)	1.1 (0.5, 1.8)	1.36 (1.13, 1.64)	0.001
**Tracheostomy, n (%)**	30 (1.2)	10,903 (1.1)	0.1 (0.0, 0.5)	1.12 (0.78, 1.59)	0.55
**CPR, n (%)**	49 (2.9)	24,303 (3.7)	0.8 (0.0, 1.7)	0.79 (0.60, 1.04)	0.09
**Any home care visits, n (%)**	1,365 (54.3)	462,270 (48.4)	5.8 (3.9, 7.8)	1.16 (1.12, 1.20)	<.0001
**Palliative care received, n (%)**	1,330 (52.9)	441,746 (46.3)	6.6 (4.7, 8.6)	1.20 (1.16, 1.24)	<.0001
				**Adjusted Regression Coefficient[a] (95% CI)**	
**Number of ED visits, mean (SD)**	1.5 (1.6)	1.8 (2.0)	0.3 (0.2, 0.4)	-0.21 (-0.26, -0.15)	<.0001
**Number of hospitalizations, mean (SD)**	1.1 (1.2)	1.2 (1.2)	0.1 (0.0, 0.1)	0.01 (-0.03, 0.05)	0.63
**Total days in the hospital, mean (SD)**	13.4 (20.7)	14.0 (21.0)	0.6 (0.0, 1.4)	0.30 (-0.46, 1.06)	0.44
**Number of ICU admissions, mean (SD)**	0.3 (0.7)	0.3 (0.6)	0.0 (0.0, 0.1)	0.03 (0.00, 0.06)	0.02
**Total days in an ICU, mean (SD)**	1.9 (7.5)	1.7 (6.5)	0.2 (0.0, 0.5)	0.29 (0.00, 0.58)	0.05
**Number of episodes of mechanical ventilation, mean (SD)**	1.3 (7.4)	1.2 (6.2)	0.2 (0.0, 0.4)	0.21 (-0.07, 0.50)	0.14
**Total number of physicians seen, mean (IQR)**	12.8 (9.4)	11.9 (8.8)	0.9 (0.6, 1.3)	1.07 (0.76, 1.39)	<.0001
**Home care visits, mean (SD)**	38.3 (81.5)	29.2 (67.9)	9.2 (6.5, 11.8)	11.25 (8.07, 14.43)	<.0001

© 2019 Wunsch H et al. *JAMA Network Open.*

CI = confidence interval; ICU = intensive care unit; ED = emergency room; SD = standard deviation; IQR = interquartile range; CPR = cardiopulmonary resuscitation

[a] adjusted for age, sex, income quintile, geographic region, Charlson comorbidity score (categorized as 0, 1-2, 3-4, and ≥ 5)

© 2019 Wunsch H et al. *JAMA Network Open.*

eTable 5. Intensity of Care Measures for Physicians Compared With Nonphysicians at the End of Life With a Diagnosis of Specific Chronic Comorbidities (Matched 1:3)[a]

	Physicians (n = 1,375)	Nonphysicians (n = 4,117)	Absolute Difference (95% CI)	Adjusted Relative Risk[b] (95% CI)	P Value
**Location of death, n (%)**					
**Home**	484 (35.2)	1,264 (30.7)	4.5 (1.7, 7.3)	1.12 (1.04, 1.22)	0.0045
**Long-term care facility**	140 (10.2)	445 (10.8)	0.6 (0.0, 2.5)	0.96 (0.81, 1.15)	0.67
**Acute care hospital (non-ICU)**	545 (39.6)	1,802 (43.8)	4.1 (1.1, 7.2)	0.92 (0.85, 0.98)	0.02
**Acute care hospital (ICU)**	163 (11.9)	448 (10.9)	1.0 (0.0, 2.9)	1.08 (0.92, 1.28)	0.35
**Other**	43 (3.1)	158 (3.8)	0.7 (0.0, 1.9)	0.80 (0.58, 1.12)	0.20
**Last 6 months of life**					
**Any ED visits, n (%)**	1,119 (81.4)	3,516 (85.4)	4.0 (1.8, 6.2)	0.96 (0.93, 0.98)	0.0009
**Any hospitalization, n (%)**	1,121 (81.5)	3,421 (83.1)	1.6 (0.0, 3.9)	0.99 (0.97, 1.01)	0.41
**Any ICU admission, n (%)**	326 (23.7)	909 (22.1)	1.6 (0.0, 4.2)	1.08 (0.97, 1.20)	0.18
**Any mechanical ventilation, n (%)**	196 (14.3)	588 (14.3)	0.0 (0.0, 2.2)	0.99 (0.86, 1.15)	0.90
**Any dialysis, n (%)**	76 (5.5)	208 (5.1)	0.5 (0.0, 1.8)	1.14 (0.89, 1.45)	0.31
**Surgical feeding tube, n (%)**	65 (4.7)	124 (3.0)	1.7 (0.6, 2.8)	1.57 (1.17, 2.11)	0.003
**Tracheostomy[c], n (%)**	17 (1.2)	45 (1.1)	0.1 (0.0, 0.8)	1.13 (0.65, 1.98)	0.67
**CPR, n (%)**	25 (2.2)	109 (3.2)	1.0 (0.0, 2.1)	0.68 (0.45, 1.05)	0.08
**Any home care visits, n (%)**	849 (61.7)	2,315 (56.2)	5.5 (2.5, 8.5)	1.11 (1.06, 1.17)	<.0001
**Palliative care received, n (%)**	868 (63.1)	2,284 (55.5)	7.7 (4.6, 10.7)	1.16 (1.11, 1.22)	<.0001
				**Adjusted Regression Coefficient[b] (95% CI)**	
**Number of ED visits, mean (SD)**	1.8 (1.7)	2.0 (1.8)	0.2 (0.1, 0.3)	-0.18 (-0.28, -0.08)	0.0004
**Number of hospitalizations, mean (SD)**	1.4 (1.2)	1.4 (1.1)	0.0 (0.0, 0.1)	0.05 (-0.02, 0.11)	0.18
**Total days in the hospital, mean (SD)**	18.1 (22.7)	17.8 (22.6)	0.4 (0.0, 1.7)	0.71 (-0.63, 2.06)	0.30
**Number of ICU admissions, mean (SD)**	0.3 (0.8)	0.3 (0.7)	0.0 (0.0, 0.1)	0.04 (-0.01, 0.08)	0.12
**Total days in an ICU, mean (SD)**	2.4 (8.6)	1.9 (6.7)	0.5 (0.1, 1.0)	0.51 (0.02, 1.00)	0.04
**Number of episodes of mechanical ventilation, mean (SD)**	1.4 (7.6)	1.2 (6.9)	0.3 (0.0, 0.7)	0.24 (-0.21, 0.69)	0.29
**Total number of physicians seen, mean (IQR)**	15.2 (9.6)	14.3 (9.0)	0.8 (0.3, 1.4)	1.09 (0.58, 1.60)	<.0001
**Home care visits, mean (SD)**	43.5 (83.9)	32.4 (70.8)	11.1 (6.6, 15.7)	11.45 (6.61, 16.28)	<.0001

© 2019 Wunsch H et al. *JAMA Network Open.*

CI = confidence interval; ICU = intensive care unit; ED = emergency room; SD = standard deviation; IQR = interquartile range; CPR = cardiopulmonary resuscitation
[a] Comorbidities include: cancer, congestive heart failure, chronic pulmonary disease, dementia, diabetes with end organ damage, peripheral vascular disease, chronic renal failure, severe chronic liver disease, or coronary artery disease as previously described [1].

[b] Adjusted for Charlson comorbidity score (categorized as 0, 1-2, 3-4, and ≥ 5)

[c]Regression model excluded Charlson comorbidity score due to sparse data.

© 2019 Wunsch H et al. *JAMA Network Open.*

eTable 6. Intensity of Care Measures for Physicians Compared With Nonphysicians at the End of Life With Cancer (Matched 1:3)

	Physicians (n = 457)	Nonphysicians (n = 1,347)	Absolute Difference (95% CI)	Adjusted Relative Risk[a] (95% CI)	P Value
**Location of death, n (%)**					
**Home**	172 (37.6)	385 (28.6)	9.1 (4.2, 13.9)	1.30 (1.13, 1.50)	0.0002
**Long-term care facility**	60 (13.1)	199 (14.8)	1.6 (0.0, 5.4)	0.89 (0.69, 1.16)	0.39
**Acute care hospital (non-ICU)**	184 (40.3)	652 (48.4)	8.1 (2.9, 13.4)	0.84 (0.74, 0.94)	0.004
**Acute care hospital (ICU)**	33 (7.2)	67 (5.0)	2.3 (0.0, 4.7)	1.46 (0.97, 2.19)	0.07
**Other**	8 (1.8)	44 (3.3)	1.5 (0.0, 3.3)	0.52 (0.25, 1.10)	0.09
**Last 6 months of life**					
**Any ED visits, n (%)**	373 (81.6)	1,193 (88.6)	7.0 (3.4, 10.5)	0.92 (0.88, 0.97)	0.0008
**Any hospitalization, n (%)**	406 (88.8)	1,229 (91.2)	2.4 (0.0, 5.5)	0.98 (0.94, 1.01)	0.20
**Any ICU admission, n (%)**	73 (16.0)	182 (13.5)	2.5 (0.0, 6.2)	1.19 (0.93, 1.51)	0.17
**Any mechanical ventilation, n (%)**	37 (8.1)	102 (7.6)	0.5 (0.0, 3.4)	1.07 (0.75, 1.53)	0.71
**Any dialysis[b], n (%)**	11 (2.4)	20 (1.5)	0.9 (0.0, 2.3)	1.62 (0.80, 3.30)	0.18
**Surgical feeding tube, n (%)**	25 (5.5)	58 (4.3)	1.2 (0.0, 3.4)	1.27 (0.79, 2.02)	0.32
**Tracheostomy[c], n (%)**					
**CPR[b], n (%)**	6 (1.5)	15 (1.2)	0.3 (0.0, 1.5)	1.21 (0.47, 3.11)	0.69
**Any home care visits, n (%)**	334 (73.1)	935 (69.4)	3.7 (0.0, 8.5)	1.05 (0.99, 1.13)	0.12
**Palliative care received, n (%)**	408 (89.3)	1,175 (87.2)	2.1 (0.0, 5.5)	1.03 (0.99, 1.06)	0.19
**Any chemotherapy, n (%)**	173 (37.9)	402 (29.8)	8.0 (3.1,12.9)	1.28 (1.13, 1.46)	<.0001
**Any radiotherapy, n (%)**	146 (31.9)	387 (28.7)	3.2 (0.0, 8.1)	1.12 (0.96, 1.30)	0.14
				**Regression Coefficient[a] (95% CI)**	
**Number of ED visits, mean (SD)**	1.8 (1.7)	2.3 (1.9)	0.4 (0.2, 0.6)	-0.41 (-0.60, -0.23)	<.0001
**Number of hospitalizations, mean (SD)**	1.6 (1.2)	1.6 (1.1)	0.0 (0.0, 0.1)	0.02 (-0.10, 0.15)	0.73
**Total days in the hospital, mean (SD)**	18.8 (20.2)	18.3 (19.4)	0.5 (0.0, 2.6)	0.61 (-1.44, 2.66)	0.56
**Number of ICU admissions, mean (SD)**	0.2 (0.6)	0.2 (0.5)	0.0 (0.0, 0.1)	0.04 (-0.02, 0.10)	0.23
**Total days in an ICU, mean (SD)**	1.5 (6.3)	1.1 (5.6)	0.5 (0.0, 1.1)	0.47 (-0.18, 1.12)	0.16
**Number of episodes of mechanical ventilation, mean (SD)**	0.8 (4.4)	0.7 (6.5)	0.1 (0.0, 0.7)	0.09 (-0.44, 0.62)	0.74
**Total number of physicians seen, mean (IQR)**	16.4 (8.6)	16.0 (8.0)	0.5 (0.0, 1.4)	0.54 (-0.33, 1.42)	0.22
**Home care visits, mean (SD)**	39.7 (64.6)	38.3 (66.6)	1.4 (0.0, 8.4)	1.49 (-5.39, 8.36)	0.67

[a] Adjusted for Charlson comorbidity score (categorized as 0, 1-2, 3-4, and ≥ 5)

© 2019 Wunsch H et al. *JAMA Network Open.*

[b]Regression model excluded Charlson comorbidity score due to sparse data.
[c] Suppression due to small cell size required as per ICES policy.

© 2019 Wunsch H et al. *JAMA Network Open.*

eFigure. Location of Death for Physicians and Nonphysicians, by Age at Death

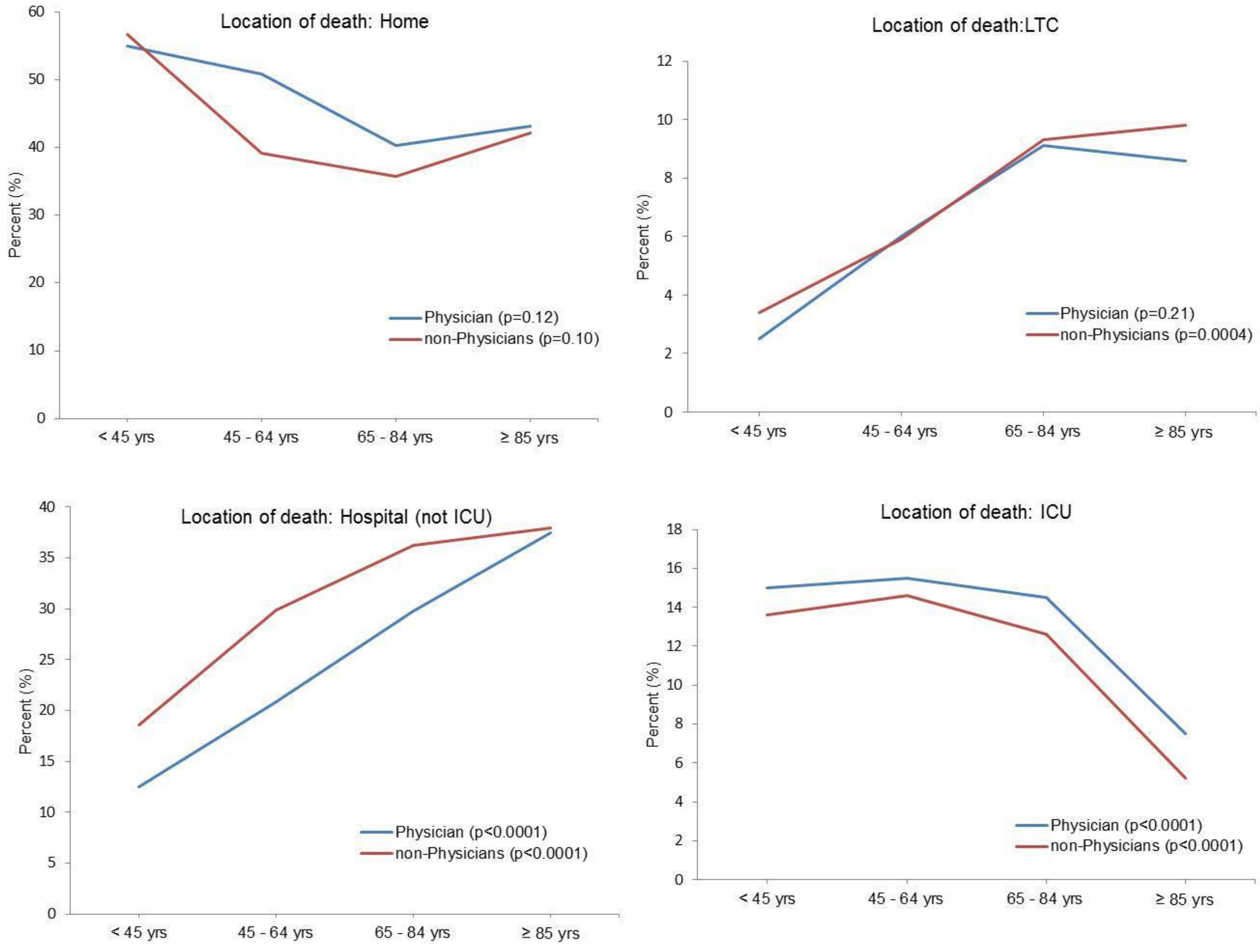

© 2019 Wunsch H et al. *JAMA Network Open.*

eReference

<jrn>1. Iezzoni LI, Heeren T, Foley SM, Daley J, Hughes J, Coffman GA. Chronic conditions and risk of in-hospital death. *Health Serv Res*. 1994;29(4):435-460. Medline:7928371</jrn>

© 2019 Wunsch H et al. *JAMA Network Open.*
